# Durable and controlled depletion of neutrophils in mice

**DOI:** 10.1038/s41467-020-16596-9

**Published:** 2020-06-02

**Authors:** Gael Boivin, Julien Faget, Pierre-Benoit Ancey, Aspasia Gkasti, Julie Mussard, Camilla Engblom, Christina Pfirschke, Caroline Contat, Justine Pascual, Jessica Vazquez, Nathalie Bendriss-Vermare, Christophe Caux, Marie-Catherine Vozenin, Mikael J. Pittet, Matthias Gunzer, Etienne Meylan

**Affiliations:** 1 Swiss Institute for Experimental Cancer Research, School of Life Sciences, Ecole Polytechnique Fédérale de Lausanne, CH-1015 Lausanne, Switzerland; 2 Laboratory of Radiation Oncology, Department of Radiation Oncology, Department of Oncology, CHUV, Lausanne University Hospital and University of Lausanne, CH-1011 Lausanne, Switzerland; 3 Swiss Cancer Center Léman, Lausanne, Switzerland; 4 University of Lyon, Université Claude Bernard Lyon 1, INSERM 1052, CNRS 5286, Centre Léon Bérard, Cancer Research Center of Lyon, 69373 Lyon, France; 5 Center for Systems Biology, Massachusetts General Hospital Research Institute and Harvard Medical School, Boston, MA 02114 USA; 6 Institute for Experimental Immunology and Imaging, University Hospital, University Duisburg–Essen, Essen, Germany

**Keywords:** Applied immunology, Cell death and immune response, Neutrophils, Innate immunity

## Abstract

Neutrophils are an essential part of the innate immune system. To study their importance, experimental studies often aim to deplete these cells, generally by injecting anti-Ly6G or anti-Gr1 antibodies. However, these approaches are only partially effective, transient or lack specificity. Here we report that neutrophils remaining after anti-Ly6G treatment are newly derived from the bone marrow, instead of depletion escapees. Mechanistically, newly generated, circulating neutrophils have lower Ly6G membrane expression, and consequently reduced targets for anti-Ly6G-mediated depletion. To overcome this limitation, we develop a double antibody-based depletion strategy that enhances neutrophil elimination by anti-Ly6G treatment. This approach achieves specific, durable and controlled reduction of neutrophils in vivo, and may be suitable for studying neutrophil function in experimental models.

## Abstract (web summary)

Anti-Ly6G or ant-Gr1 antibodies are commonly used to deplete neutrophils in vivo. Here the authors provide mechanistic insight into why these approaches may not specifically or durably reduce the number of neutrophils in mice, and also present a new method that overcomes these limitations to have potentially wide applicability in experimental studies.

## Introduction

With an estimated daily production of 1 billion cells/kg in the bone marrow, neutrophils are the most prevalent immune subset found in human blood^1^. Neutrophilia was shown to correlate with a myriad of pathological situations, including auto-immunity, chronic infection or inflammation, wound healing and cancer^2–5^. To decipher whether neutrophils contribute to these pathological events either positively or negatively, anti-neutrophil antibodies have been extensively used to deplete these cells in vivo^6–9^. However, the two antibodies available, namely anti-Ly6G clone 1A8 (specific for the Ly6G protein) and anti-Gr1 clone RB6-8C5 (that recognizes both Ly6G and Ly6C), suffer some limitations.

Anti-Gr1 was the first available antibody to deplete neutrophils. It recognizes Ly6G, the most widely used marker of neutrophils, and Ly6C that is additionally expressed on monocytes, macrophages, T-cell subsets, eosinophils and small-vessel endothelial cells^10^. Anti-Gr1 is a rat IgG2b that induces cell death through complement-mediated membrane-complex attack^11^.

Unlike anti-Gr1, anti-Ly6G has the advantage to target neutrophils specifically^12^. However, it is a rat IgG2a that induces a Fc-dependent opsonization and phagocytosis of targeted cells^13^. Anti-Ly6G has commonly been associated with a lower efficiency than anti-Gr1 to deplete neutrophils, sometimes resulting in contradictory experimental results^14^.

Both antibodies display a short window of efficacy, as a “rebound” has been typically described after a few days of treatment both in infectious and cancer models^6,15^. This rebound may rely on bone marrow response to peripheral loss^16^ (compensation), anti-rat antibody production by the treated mice^15^ (mitigation), limited depletion in peripheral tissue^16^ (low bioavailability) or induction of neutrophil production in the spleen^16^ (extra-medullary granulopoiesis).

Using an in vivo radioactive labelling methodology, Pillay et al.^17^ estimated that mouse neutrophils mature during 2.3 days (5.8 days in human) within the bone marrow, before they get released and circulate for 0.75 days (5.4 days in humans). The results regarding the bone marrow maturation time obtained with this approach corresponded to previous studies^18,19^, however the peripheral transit time was 10 times greater than previously estimated for human neutrophils^20–22^. According to Pillay et al., these latter approaches required ex vivo labelling, which may have impacted their activation status and circulating time in blood. This explanation remains controversial, as other authors have suggested alternative interpretations of their results^23^. Although neutrophil peripheral lifespan remains debated, these studies indicate that the bone marrow maturation transit time under non-inflamed physiological conditions is greater than the circulating one.

Neutrophil production rate increases by a factor of 10 upon acute stress, stimulation with G-CSF or peripheral neutrophil loss^1^. Although the observed phenotypes obtained with depletion strategies have always been attributed to the loss of neutrophils, with such tremendous dynamics, it appears important to further characterize the residual neutrophil fraction and assess whether it carries functionality.

In this work, we postulate that the residual neutrophil fraction or the nonspecific targeting can bias data interpretations from anti-Ly6G or anti-Gr1 based experiments and propose an optimized, standardized and neutrophil-specific depletion strategy to alleviate this ambiguity.

## Results

### Intracellular Ly6G staining overcomes surface unavailability

**Fig. 1:**
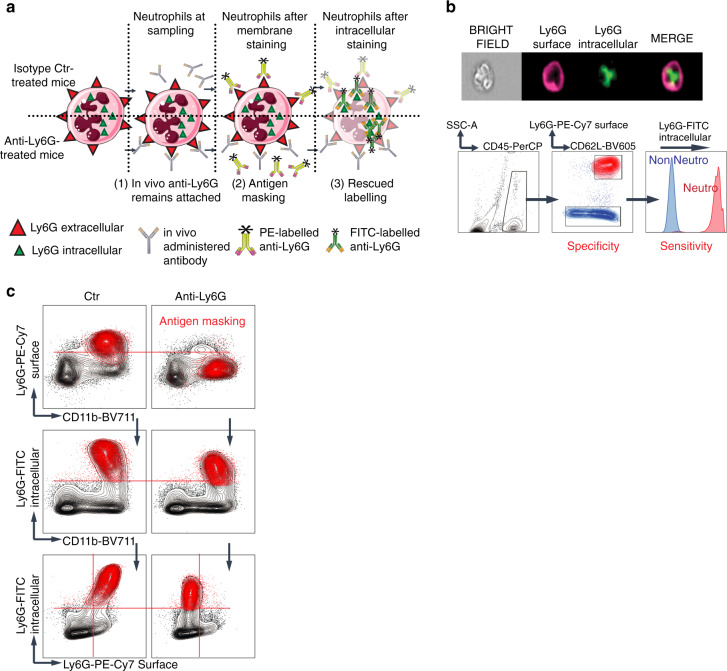
Intracellular Ly6G staining overcomes surface Ly6G unavailability. **a** Schematic representation of the antigen masking related issue. Upon anti-Ly6G treatment, the in vivo delivered antibody remains bound to the surface antigen even after sampling, impairing fluorochrome-labeled additional binding. In contrast, the intracellular antigen remains available. **b** (top) Representative image acquired with ImageStream of a neutrophil where the Ly6G antigen has been successively and specifically stained on the membrane then in the intracellular compartment. (down) Representative flow cytometry analysis emphasizing the specificity and sensitivity of the intracellular Ly6G staining. **c** Mice were treated for 24 h with an isotype control (left) or anti-Ly6G (right) antibody, after which the bone marrows were sampled. CD45^+^CD11b^+^Ly6G^intra+^ neutrophils are shown in red. In the anti-Ly6G-treated group, cells were not detectable using the extracellular staining because of the antigen masking.

A known yet often overruled issue in cell depletion strategies is that of antigen masking, whereby an antibody used in vivo will prevent the binding of the same antibody if used to detect the target cells. Specifically, the use of fluoro-labelled anti-Ly6G in flow cytometry staining results in low sensitivity and false negative measures. We hypothesized the intracellular amount of Ly6G would be sensitive enough to detect neutrophils independently from the treatment-induced limitations (Fig. 1a). First, we validated with the ImageStream technology that both inner and outer Ly6G could be detected independently with an equal sensitivity and specificity on untreated mouse neutrophils, defined as CD45^+^Ly6G^+^CD11b^+^CD62L^+^ in the bone marrow (Fig. 1b). Then we validated our staining procedure on anti-Ly6G treated mice (Fig. 1c) and concluded that intracellular staining is a highly sensitive and specific mean to detect neutrophils upon depletion treatment despite membrane antigen masking. Other gating strategies, including Ly6C and side scatter (SSCa) can be used and have been reported in the literature, exemplified here using Catchup^IVM-red^ (*Ly6g*^Cre-tom/wt^; *ROSA26*^Rtom/wt^, C57BL/6) mice^24^ whose neutrophils can be specifically identified based on tdTomato expression, however SSCa lacks specificity (Supplementary Fig. 1a–c).

### Neutrophils upon anti-Ly6G have surface Ly6G paucity in vivo

**Fig. 2:**
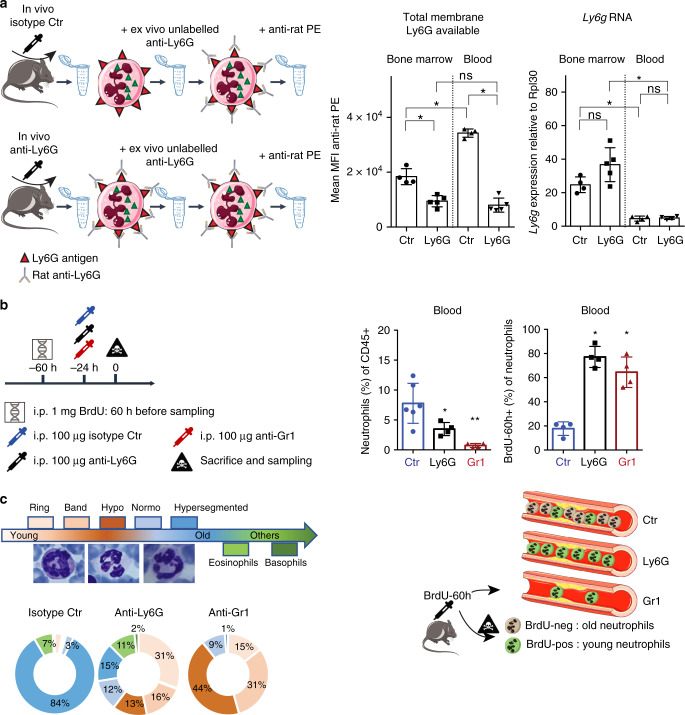
Newly synthesized neutrophils upon anti-Ly6G have membrane Ly6G paucity. **a** Old (>20 weeks) C57BL/6 mice were treated with anti-Ly6G 50 μg daily for 9 days. After sampling, bone marrow and blood cells were re-incubated with 10 μg of anti-Ly6G to saturate the Ly6G antigen, and subsequently stained with a donkey anti-rat PE-labelled antibody to measure the whole amount of Ly6G antigen at the surface of neutrophils. MFI, mean fluorescence intensity. RNA levels for *Ly6g* on purified neutrophils from the same mice is also shown in the right panel. *n* = 4 control (Ctr) and 5 anti-Ly6G mice. **b** (left) Setting for experiments performed on C57BL/6 mice of >20 weeks. (right) Prevalence of CD45^+^CD11b^+^Ly6G^intra+^ neutrophils among total CD45^+^ immune cells and their respective BrdU-60h incorporation. **c** Neutrophil segmentation, which correlates with neutrophil aging, was analyzed on Giemsa-stained blood smears. *n* = 5 control (Ctr) and 4 treated mice. All the results from (**b**, **c**) were acquired from the same blood samples. *p < 0.05, from Mann–Whitney test; error bars represent s.d. Source data are provided as a Source Data file.

As the mean fluorescence intensity (MFI) for Ly6G remained lower on anti-Ly6G/Gr1 treated mice despite adequate labelling, we asked whether this loss of signal was due to some technical labelling issue or was a real biological measure. Mice were treated for 9 days with anti-Ly6G and sacrificed. After bone marrow and blood collection, cells were resuspended in 10 μg of anti-Ly6G to saturate the membrane Ly6G antigen and subsequently stained with an anti-rat PE labelled antibody. In this setting, there is no antigen masking by definition as all the anti-Ly6G bound at the membrane will subsequently be revealed by the anti-rat PE antibody. We found a lower MFI for Ly6G upon anti-Ly6G treatment revealing a lower amount of membrane Ly6G at the protein level. On the same samples, previously split for RNA analysis, we found a trend toward increased *Ly6g* gene expression in the bone marrow (Fig. 2a and Supplementary Fig. 2a), while blood neutrophils had a low level of *Ly6g* mRNA expression in both treatment conditions suggesting the gene is mostly transcribed within the bone marrow. We conclude the residual fraction of neutrophils upon anti-Ly6G treatment has low level of membrane antigen.

### Residual neutrophils are newly made cells

We sought to clarify whether anti-Ly6G is less efficient than anti-Gr1. Old C57BL/6 mice, which are the most refractory strain to anti-Ly6G (Supplementary Fig. 3a–d), were injected with BrdU 36 h before a single anti-Ly6G or anti-Gr1 injection and sampled 24 h after antibody delivery (Fig. 2b). Neutrophil prevalence in blood was reduced by 50 and 90% in the anti-Ly6G and anti-Gr1 groups, respectively (Fig. 2b and Supplementary Fig. 2b). In each treatment condition the amount of BrdU-60h positive cells in the blood raised from 20 to >60% (Fig. 2b and Supplementary Fig. 2b). Blood smear analysis confirmed residual neutrophils have lower amounts of nuclear segmentation in both treated groups, especially upon anti-Gr1 (Fig. 2c). We conclude that both anti-Ly6G and anti-Gr1 are efficient to remove neutrophils because the remaining circulating cells are newly made cells. However, this effect does not equivalently impact neutrophil prevalence in blood. We hypothesized the killing celerity of anti-Ly6G was inferior to neutrophil production in the bone marrow, because neutrophil prevalence is the product of cellular production rate (incidence) and lifespan.

### Killing celerity optimization with anti-Ly6G isotype switch

**Fig. 3:**
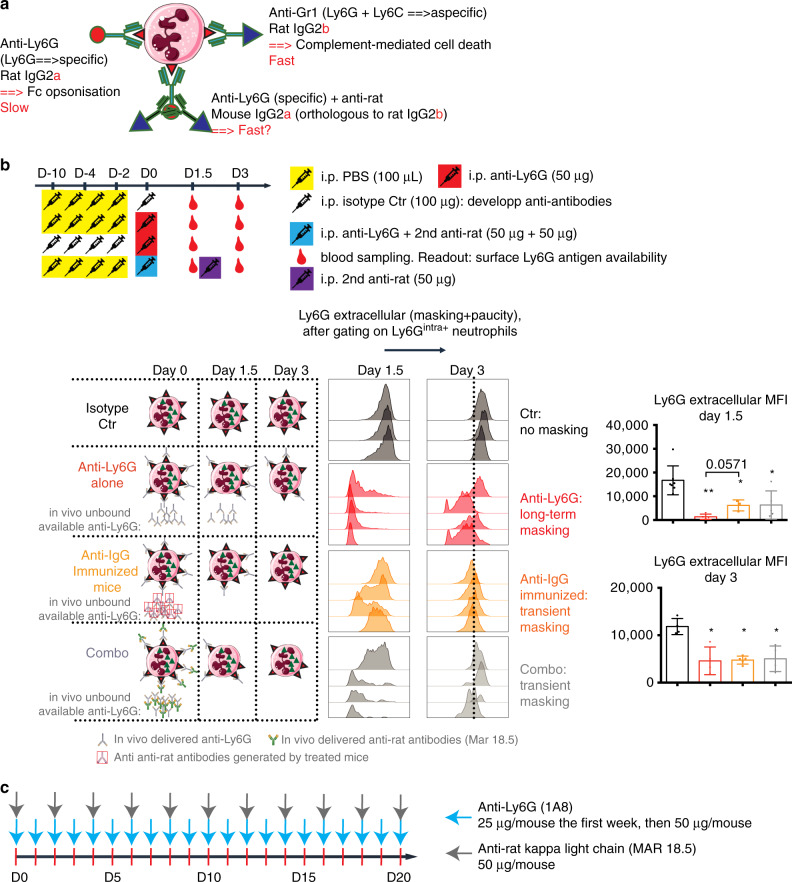
Rationale for an optimized anti-Ly6G-based “Combo” depletion strategy. **a** Scheme introducing the “isotype switch” strategy to enhance the anti-Ly6G killing celerity. **b** Experimental plan to evaluate the impact of anti-antibodies on the ability of anti-Ly6G to bind the Ly6G antigen. Neutrophils from blood were gated as CD45^+^CD11b^+^Ly6G^intra^^+^. The MFI of Ly6G extracellular was then measured. One can see that both anti-IgG immunized mice or mice treated with anti-Ly6G + anti-rat (MAR 18.5) antibodies have more Ly6G antigens available 1.5 days after anti-Ly6G injection. **c** According to the data obtained in (**b**), the ideal scheme for double antibody-based neutrophil depletion requires a daily injection of anti-Ly6G. *p < 0.05, **p < 0.01 from Mann–Whitney test; error bars represent s.d. 5–7 control (Ctr) and 4–5 treated mice were used per group. Source data are provided as a Source Data file.

Anti-Gr1 recognizes more antigens on the surface of neutrophils (Ly6G + Ly6C) and has a different isotype than anti-Ly6G. Anti-Ly6G is a rat IgG2a isotype, orthologous to the mouse IgG1 heavy chain, which has been shown to mediate neutrophil killing through Fc-dependent macrophage opsonization^13^. Anti-Gr1 is a rat IgG2b, orthologous to the mouse IgG2a heavy chain, expected to work through both classical and alternative complement pathways^11^. We reasoned that the amount of membrane-bound antibody and the difference in isotype may have an impact on the killing celerity of each antibody. To investigate this, we combined the anti-Ly6G with a mouse IgG2a anti-rat antibody, each of which should recognize two anti-Ly6G molecules and mirror an “isotype switch” (Fig. 3a).

### Standardization of anti-Ly6G based turnover induction

First, we tested whether the presence of an anti-rat secondary antibody neutralizes the anti-Ly6G. We injected mice with 100 μg of anti-rat, followed two hours later by an anti-Ly6G injection (reverse (R)-Combo). After 24 h, we observed a strong depletion of neutrophils in blood and lung, indicating that anti-Ly6G manages to reach its target despite the presence of anti-rat antibodies (Supplementary Fig. 4). Next, we used the antigen masking issue as an advantage to indirectly measure the bioavailability of injected anti-Ly6G. 1.5 days after a single dose, neutrophils could be detected using intracellular Ly6G staining, while the membrane antigen was completely masked (Fig. 3b). The masking was partial 3 days after injection, suggesting the anti-Ly6G concentration in treated mice was too low to fully recover the corresponding epitope at the surface of neutrophils.

Mice treated with anti-Ly6G or a relevant isotype control develop anti-rat antibodies in the course of one week (Supplementary Fig. 5). These anti-antibodies cross-react with both primary antibodies, suggesting they target a shared antigen of the primary isotypes (Supplementary Fig. 5) and are not anti-idiotypic. We immunized mice with an isotype control 10 days before anti-Ly6G administration, to experimentally mimic the effect of an anti-rat antibody upon anti-Ly6G. In this setting, the epitope availability was rescued much faster after anti-Ly6G delivery (Fig. 3b). We conclude that anti-[anti-rat] antibodies accelerate the turnover of in vivo-delivered antibodies but do not impair the antigen recognition. Next, we combined anti-Ly6G with the anti-rat antibody at isodoses. Corroborating the results in pre-immunized mice, 1.5 day later, residual neutrophils displayed partial masking, suggesting the Ly6G epitope was partially occupied by the antibody. Summarizing our results, anti-Ly6G should be injected daily to encompass a lower half-life that is artificially reduced in the combination setting and biologically reduced by the occurrence of anti-antibodies developed by treated mice. Additional experiments allowed us to diminish the dose of anti-Ly6G to 25 μg to fully mask neutrophils even when mice were more than 30 g of weight. To spare a daily double injection on mice, we deliver the secondary antibody only once every other day according to our final protocol named “Combo” (Fig. 3c).

### Combo specifically and durably reduces neutrophil prevalence

**Fig. 4:**
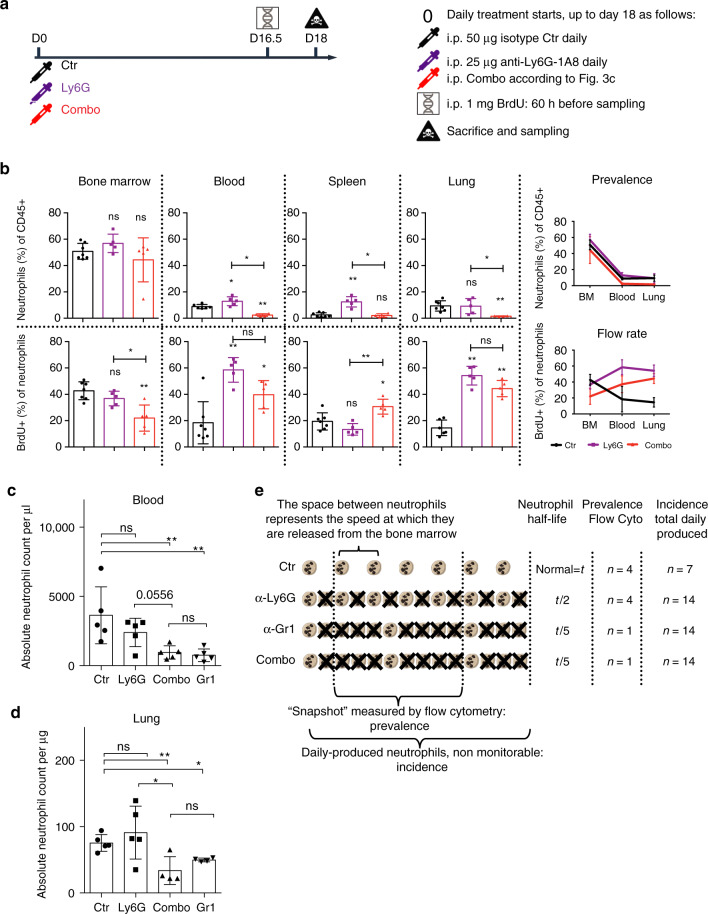
Combo induces neutrophil turnover and reduces their prevalence. **a** Old C57BL/6 mice (>20 weeks) were treated with isotype control antibody (Ctr), anti-Ly6G or the combination strategy (Combo) for 18 days. 60 h before sampling, mice were injected once with 1 mg of BrdU intraperitoneally. **b** Neutrophil prevalence among all immune cells was assessed in bone marrow, blood, spleen and lung while the impact on their dynamic flow rate was measured with BrdU-60h labelling. Both anti-Ly6G and Combo treatment elicit a shift of BrdU neutrophils from the bone marrow stock to the peripheral tissue, but only the Combo treatment is “fast” enough to also reduce neutrophil prevalence. BM, bone marrow. **c**,**d** Validation of protocol efficacy with Trucount flow cytometry analysis in (**c**) blood and (**d**) lung tissue. **e** Scheme representing neutrophil counts in the circulation from a dynamic perspective, with the impact of increased flow rate on the global prevalence of neutrophils depending on the killing celerity of the depletion strategy. *p < 0.05, **p < 0.01 from Mann–Whitney test; error bars represent s.d. **b** *n* = 7 control (Ctr) and 5 treated mice per group. **c**,**d** *n* = 5 mice per group. Source data are provided as a Source Data file.

The ability of anti-Ly6G to reduce neutrophil prevalence is strain- and age-dependent and might be influenced by a tumoral status (see Supplementary Fig. 3). We challenged our protocol for 18 days in old C57BL/6 mice, the most difficult situation to achieve depletion. At the end of the experiment, the prevalence of neutrophils in the bone marrow was maintained (Fig. 4a, b). We found a significant lowering of the BrdU positive neutrophils in the bone marrow while this proportion was increased in the blood and tissue, suggesting neutrophils were faster mobilized from the bone marrow to the periphery upon treatment. Only Combo drastically reduced neutrophil prevalence in blood and lung tissue. In spleen, where basal neutrophil levels are inherently low, we did not detect a further reduction with Combo. Anti-Ly6G alone did not reduce neutrophil prevalence, even if BrdU positive neutrophils in the bone marrow, blood and lung suggested an increased mobilization from the bone marrow to the periphery (Fig. 4a, b). Furthermore, we measured an equivalent induction of granulopoiesis^25^ with both strategies as soon as after one day of treatment (Supplementary Fig. 6a). To value the use of a combination approach instead of anti-Gr1 or anti-Ly6G alone, we verified that other Ly6C^+^ immune cells were unaffected by the treatment using regular or Trucount flow cytometry (Supplementary Figs. 6b–e and 7). We also validated using Trucount flow cytometry that Combo but not anti-Ly6G alone reduced, comparably to anti-Gr1, neutrophil counts per μl of blood or per μg of lung tissue (Fig. 4c, d and Supplementary Fig. 2c). Finally, we verified that Combo did not affect classical blood parameters, such as absolute white and red blood cell and platelet (Plt) counts, % of red blood cell hemoglobin, mean corpuscular volume (MCV) or platelet width (Supplementary Fig. 8). Altogether, these results confirm that anti-Ly6G is efficient to enhance neutrophil turnover but is not fast enough to mitigate the bone marrow response and reduce peripheral neutrophil counts (Fig. 4e). Prevalence reduction can only be achieved to an equivalent level as anti-Gr1 with Combo. This protocol will thus allow to isolate Ly6C-mediated effects of anti-Gr1.

## Discussion

In this study, we propose a standardized and optimized anti-Ly6G based methodology to reduce neutrophil prevalence specifically both in circulating blood and tissue of interest. To do so, we showed that residual neutrophils upon anti-Ly6G are newly made cells as opposed to non-depleted cells, and display low amount of membrane Ly6G, which limits the whole amount of anti-Ly6G antibody at the surface of circulating neutrophils. Next we used an intracellular Ly6G-based staining method to detect neutrophils with high sensitivity and specificity while using membrane competition as an advantage to measure the ability of in vivo delivered anti-Ly6G to “find its target” at the surface of neutrophils. After we understood the kinetics of anti-Ly6G distribution and clearance, we proposed a double antibody-based method to efficiently reduce the neutrophil prevalence in a long-term way.

In 2015, a news feature pointed that the misuse of antibodies may be a major source of the “science non-reproducibility crisis”^26^. Resources to target antigens with in vivo delivered antibodies for research are limited: as opposed to the ones used in clinical settings, the majority of antibodies available are from rat isotypes. Consequently, anti-rat antibodies developed by recipient mice are expected to occur. We learned from the clinics that antibodies from a xenogenic source have a short half-life (muromonab, murine anti-CD3 in human: half-life 18 h^27^). Having a short half-life does not necessarily imply antibodies are neutralized: antibodies developed against the non-idiotypic fraction are expected to generate immune complexes and increase the speed of degradation of delivered antibodies (reduced bioavailability, common), while antibodies developed against the idiotypic fraction are expected to neutralize them by disabling the antigen-antibody interaction^28^ (reduced efficiency, rare). In our setting, we found a cross-recognition of anti-antibodies against both anti-Ly6G and its relevant isotype control, suggesting the response was targeting heavy chains sharing similar properties. Yet, because the treatment remained efficient in the combination setting, it also indicates that the anti-Ly6G was reaching its target. It implicates that any delivery of rat isotype antibodies in immunocompetent mice should be adjusted (starting from days 4 to 7) on a daily basis. For instance, injecting mice twice weekly may be sufficient the first week, but would result in “non-treated” periods of 5 days out of seven, which strengthens the need for optimized and standardized strategies. This fact may apply to many other antibody-based strategies.

In a previous work, we had proposed to inject anti-Ly6G less frequently and with a one-day delay before the anti-rat administration^29^. However, this approach was not long-lasting and insufficient to reduce the neutrophil prevalence in tissue. A one-day delay may be too long to circumvent the bioavailability issue, especially in the context of a secondary anti-rat antibody. Thus, we recommend to use our optimized “Combo” developed in the present study.

Anti-anti-Ly6G IgMs are not responsible for the resistance to anti-Ly6G depletion. It was indeed shown that neutrophil rebound still occurs after 4 days of anti-Gr1 treatment despite adjuvant B cell depleting strategies mediated by chemotherapy, as well as with daily anti-Gr1 injection to circumvent the bioavailability issue^15^. Enhanced granulopoiesis has been previously reported with both anti-Ly6G/Gr1^16^ and therefore proposed as a mechanism of resistance to anti-Ly6G/Gr1 mediated depletion. However, anti-Ly6G has also been shown to be highly but transiently efficient in metastatic tumor models with high G-CSF driven granulopoiesis^6^. This implies enhanced granulopoiesis is not enough to intrinsically limit the anti-Ly6G efficacy. We found circulating cells upon anti-Ly6G had low levels of surface-bound anti-Ly6G, which coincides with a down-regulation of the *Ly6g* transcription that occurs upon neutrophil exit from the bone marrow. There are several predicted causes for this membrane paucity of Ly6G. (1) As Ly6G is expressed all along neutrophil maturation, the acceleration of neutrophil flow rate, as measured by the BrdU kinetics, may result in neutrophil exit before they reach full membrane maturation; (2) the selective pressure of the anti-Ly6G antibody may preferentially remove Ly6G^high^ cells; (3) anti-Ly6G ligation may induce antibody internalization with no recycling of the Ly6G antigen; and (4) circulating FcγR positive cells from a mono-granulocytic lineage may interact with the Ly6G/anti-Ly6G complex. Independently from the cause, the peripheral membrane paucity for Ly6G is likely to reduce the efficacy of anti-Ly6G.

The dynamics of neutrophil production and speed of differentiation are remarkable and respond to the need of constantly refurnishing a short-lived highly prevalent cell population^1^. Additionally, it has recently been shown that neutrophils infiltrate multiple tissues where they instruct homeostatic and pathological conditions by contributing to circadian gene regulation^30^, which strengthens the need to “take time seriously” to appreciate neutrophil physiology^31^.

It is important to implement these dynamics to fully interpret anti-Ly6G, anti-Gr1 and Combo experiments. The Ly6G antigen is expressed late in the neutrophil ontogeny, and is not found on neutrophil progenitors or during the mitotic phase of the lineage^32^. Accordingly, antibody-based strategies target differentiated non proliferative cells, but not the initial source of neutrophils that is further activated to compensate the peripheral loss^16^. After initial efficacy, none of these antibody strategies managed to mitigate the neutrophil pool within the bone marrow. The residual neutrophils from all these strategies showed an increased turnover, as they were found to be “younger”. The differential effect on peripheral prevalence, which is the product of cellular lifespan to cellular flow rate, in absence of bone marrow depletion, thus most likely reflects a differential killing celerity. We chose the term “young” instead of “immature” to define residual circulating neutrophils, as the latter possibly carries a connotation of dysfunction, while poorly segmented young neutrophils in the context of infection for instance are functional and necessary to mitigate infection^33^. We also propose to be careful about the term “depletion” and emphasize the “enhanced turnover”, because the residual neutrophil fraction might be an important component in antibody-based experiments. For example, it would be interesting to evaluate if this residual fraction displays equivalent granular composition, as their maturation is “age” dependent^31^. This is a critical difference with chemotherapy-induced neutropenia that results from a loss of progenitors^34^, leading to agranulocytosis. Importantly, in this setting, mice infected with various pathogens die in the course of a week, while comparable experiments using anti-Ly6G/anti-Gr1 rarely induce lethality, but rather a delay in pathogen clearance^35–38^. This may indicate that the residual fraction observed upon antibody-based depletion is sufficient to mitigate the severity of infection and thus be functionally relevant.

## Methods

### Mouse importation and housing conditions

All mouse experiments complied with all relevant ethical regulations for animal testing and research. Experiments from the Ecole Polytechnique Fédérale de Lausanne were performed with the ethical approval of the Veterinary Authority of the Canton de Vaud, Switzerland (license number VD2391). Mouse experiments at Massachusetts General Hospital were performed according to approved IACUC guidelines. Wild type C57BL/6 mice were purchased from Jackson Laboratory. KP1.9 tumors were injected intravenously as previously described^39^. Mouse experiments performed at the Cancer Research Center of Lyon, Centre Léon Bérard, Lyon, France were approved by the local Animal Ethic Evaluation Committee (CECCAPP: C2EA-15) and authorized by the French Ministry of Education and Research. Animals were maintained in a specific pathogen free (SPF) animal facility AniCan platform.

### In vivo antibody-based treatments

Anti-Ly6G (clone 1A8, #BP0075-1), anti-Gr1 (clone RB6-8C5, #BE0075), anti-rat Kappa immunoglobulin (clone MAR 18.5, #BE0122) and corresponding isotype controls (#BP0290 and #BP0089) were all purchased from Bio X Cell and injected intraperitoneally following the schemes and dosage that are presented in the Figures or legends. When mice were sequentially injected with two antibodies, we respected 2 h delay between injections.

### Flow cytometry

For flow cytometry analysis, single cell suspension was obtained after tissue macro-dissection, following a procedure described previously^40^. All acquisitions were performed using the LSRII SORP (Becton Dickinson), a 5-laser and 18-detector analyzer at the EPFL Flow Cytometry Core Facility. Data analyses were performed using FlowJo X (FlowJo LLC ©). Trucount flow cytometry experiments were performed on 30 μl of total blood directly stained with antibodies washed into 2 ml of PBS then complemented with 25 μl of countBright absolute counting beads (C36950 Invitrogen) and resuspended into 2 ml of PBS before acquisition on a Attune NxT Flow Cytometer (ThermoFisher Scientific).

### Combined intra versus extracellular staining for Ly6G

Samples were collected and surface stained with classical methods of flow cytometry labelling. After 3 washes, cells were fixed and permeabilized using the FoxP3 staining kit from eBioscience (#00-5523-00). Then, cells were incubated with anti-Ly6G or anti-Gr1 antibodies or adequate isotype control labelled with distinct fluorochromes from the surface staining in the permeabilization buffer for 15 min at 4 °C. Intracellular staining dilution should be twice less than membrane staining. Cells were washed twice in permeabilization buffer and fixed again for 5 min at room temperature. Cell pictures were acquired using an ImagestreamX Mark II.

### BrdU injection and staining procedure

A concentration of 1 mg of BrdU (BD-552598, BD Biosciences) was injected into mice intraperitoneally at 10 mg ml^−1^. BrdU labelling was performed according to the provider’s protocol. Labelled samples were acquired within 48 h.

### Semi-quantitative ELISA

Serum of treated mice was collected after centrifugation of the collected blood (10 min 10,000 RPM). The semi-quantitative ELISAs were performed with the ab223588 kit (Abcam), but 96 well plates were coated with serum diluted at 1/25 with PBS.

### Gene expression analysis

RNA for real-time PCR was extracted using TRIzol (15596018, ThermoFisher Scientific). RNA (1 μg) was used for reverse transcription using the High-Capacity cDNA Reverse Transcription Kit (4368814, ThermoFisher Scientific). Real-time PCR was done using Taqman universal PCR master mix (4324018, ThermoFisher Scientific) and Taqman probes for the following genes: *Ly6g*: Mm04934123_m1; gene expression level normalization gene *Rpl30*: Mm01611464_g1 (ThermoFisher Scientific). Data were presented as Δ*C*_T_ values.

### Survey of the scientific literature

We surveyed the scientific literature over a one-year period (April 2017 to March 2018) and found forty-five publications using the key words “neutrophils depletion” on PubMed, in which the authors reported efficient neutrophil depletion. One-third of these publications used anti-Gr1 and two-thirds anti-Ly6G. From each study, we noted the method used to validate neutrophil depletion, or if no validation was shown.

### Statistical analysis

All results are presented as mean ± s.d. or s.e.m. as indicated. Statistical analysis was performed using nonparametric Mann–Whitney test using Prism 6 software. Statistical significance is indicated as ∗*p* < 0.05, ∗∗*p* < 0.01, ∗∗∗*p* < 0.001, ∗∗∗∗*p* < 0.0001, or ns (not significant).

### Reporting summary

Further information on research design is available in the Nature Research Reporting Summary linked to this article.

## Supplementary information

Supplementary Information

Reporting Summary

## Data Availability

All relevant data are available in the main text, Source Data file and supplementary information, or upon reasonable requests to the authors.

## Source Data

Source Data
