# Supplementary material for: Durable and controlled depletion of neutrophils in mice
Source: Nat Commun. 2020 Jun 2;11:2762. doi: 10.1038/s41467-020-16596-9 (PMC7265525; doi:10.1038/s41467-020-16596-9)
Supplement: Supplementary file 1 — Supplementary Information [file 41467_2020_16596_MOESM1_ESM.pdf]

Supplementary Information

Durable and controlled depletion of neutrophils in mice

Boivin et al.

Supplementary Figures

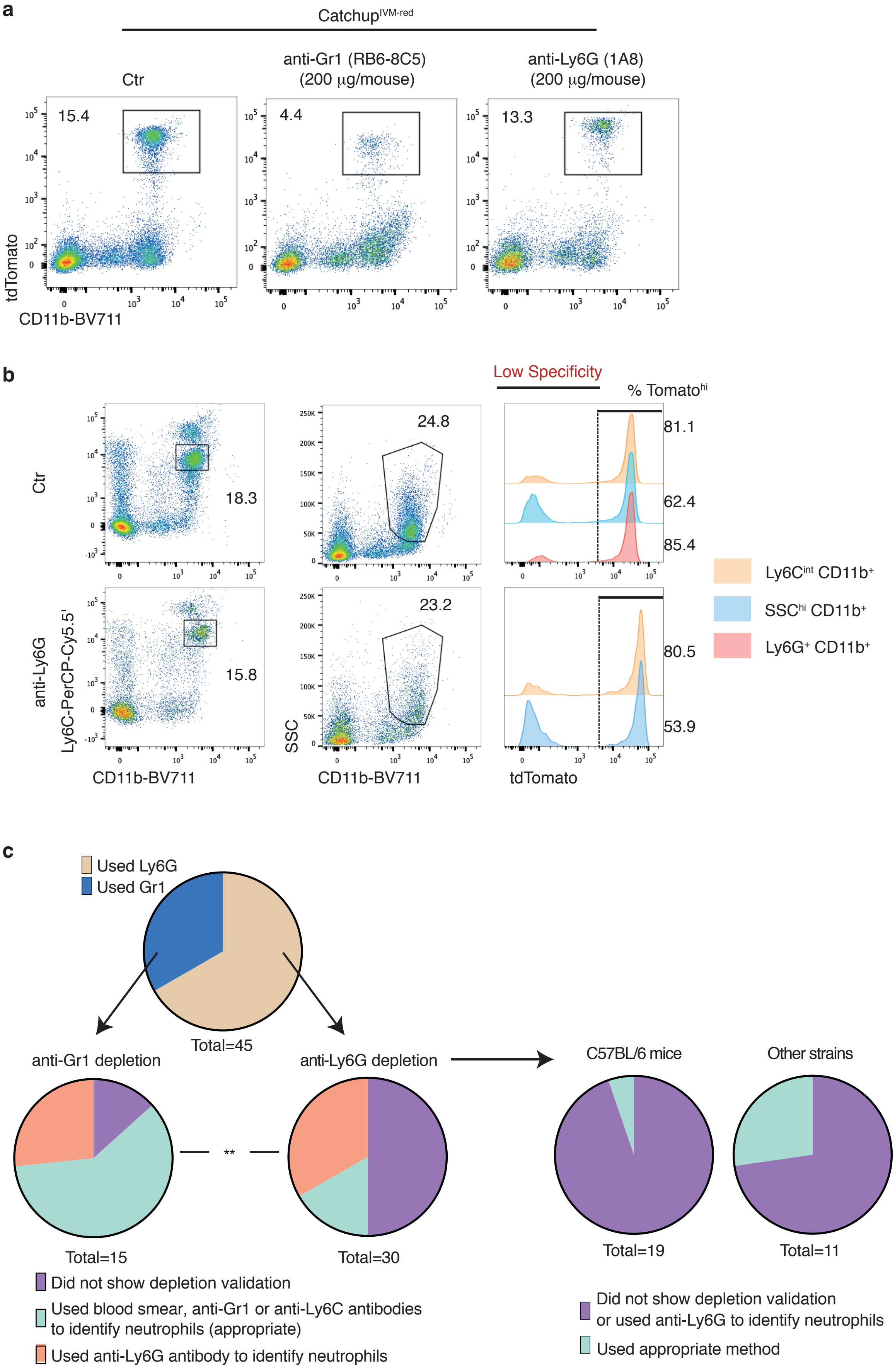

Supplementary Figure 1. SSC and Ly6C can be used to overcome the antigen masking issue but SSC lacks specificity. **a**. (left) Flow cytometry plots showing neutrophil proportion identified as CD11b$^+$tdTomato$^+$ cells among CD45$^+$ cells in 14 weeks old Catchup$^{IVM\text{-}red}$ mice treated with 200 µg of control (Ctr: clone 2A3), anti-Gr1 or anti-Ly6G antibody 24 hours before blood sampling. **b**. (left and middle) CD11b with SSC-A- or Ly6C-based alternative gating strategies that are commonly used to avoid the antigen masking issue. (right) Based on tdTomato expression from Catchup$^{IVM\text{-}red}$ mice, the right panel presents a representative evaluation of the specificity of the methods. **c**. (up) Literature analysis reporting the use of anti-Ly6G or anti-Gr1 to deplete neutrophils (total = 45 studies). (lower left) Strategies used in the papers to demonstrate neutrophil depletion efficacy when anti-Gr1 or anti-Ly6G were used. ** $p<0.01$ using Chi-scare test. (lower right) Proportion of publications using the anti-Ly6G antibody to deplete neutrophils in C57BL/6 mice (n=19) or other strains (n=11), and associated depletion validation.

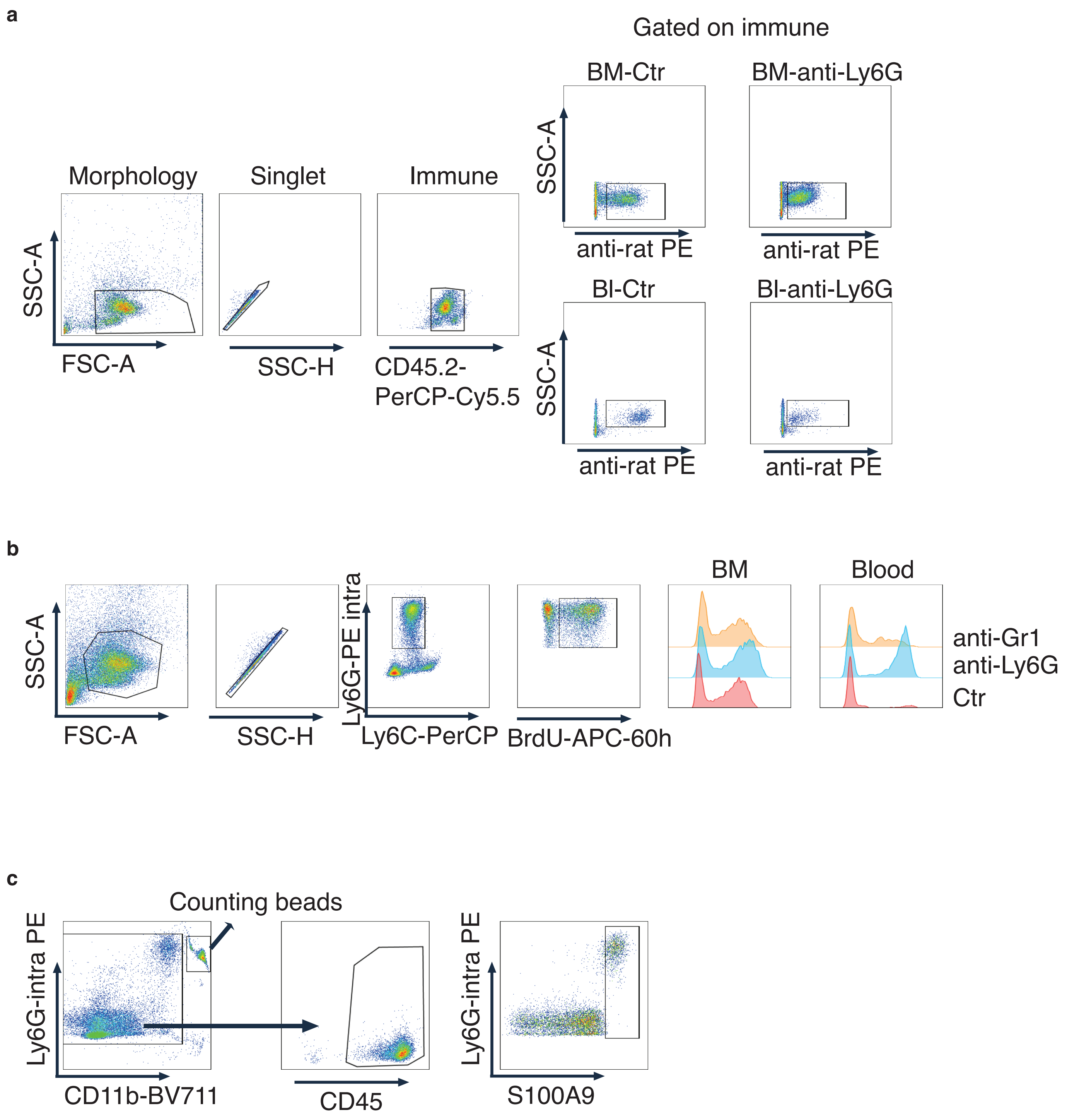

Supplementary Figure 2. Gating strategies used in the study.
a. Gating strategy used to analyze Ly6G protein and mRNA expression presented in Fig. 2a. BM, bone marrow; Bl, blood. **b**. Gating strategy used to monitor neutrophil prevalence or the proportion of BrdU⁺ neutrophils shown in Fig. 2b. **c**. Gating strategy used for Trucount flow cytometry reported in Fig. 4c and 4d.

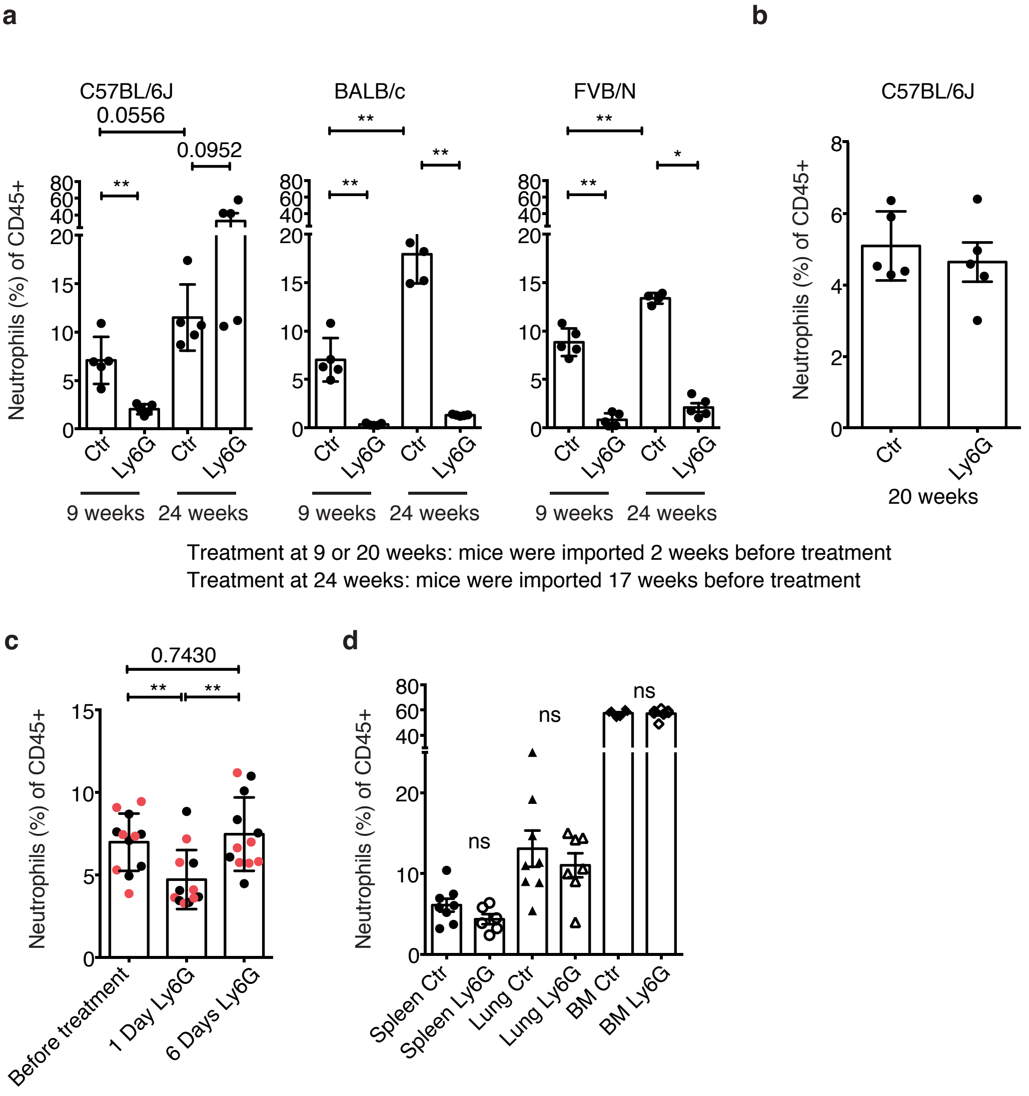

Supplementary Figure 3. Residual neutrophil prevalence upon anti-Ly6G treatment depends on mouse strain and age.

a. C57BL/6J, BALB/c and FVB/N mice imported from Charles River and treated with anti-Ly6G or control (Ctr) antibody (200 μg) for 24 hours at 9 weeks and 24 weeks of age and housed respectively during 2 or 17 weeks in the EPFL animal facility. n=4-5 mice per condition. **b**. 18 weeks old C57BL/6J mice from Charles River were housed for two weeks in the EPFL animal facility, then treated as in (**a**). **c**. Histogram shows percentage of neutrophils in blood before and at day one or day six of treatment with 150 μg of anti-Ly6G injected daily in C57BL/6J mice produced in house (black dots) or imported from Charles River (red dots). Raw flow cytometry files were provided by C. Caux's lab (INSERM, Centre Léon Bérard, France). This experiment is representative of 13 independent ones, where anti-Ly6G was tested at different concentrations (from 100 μg to 800 μg) and at different frequencies (daily to weekly). **d**. Histogram shows percentage of neutrophils in spleen, lung and bone marrow (BM) from lung tumor cell-transplanted C57BL/6J mice treated with 500 μg of anti-Ly6G (n=7) or control antibody (Ctr n=6) every two/three days over thirteen days. Tail vein $Kras^{G12D/WT}$; $p53^{Flox/Flox}$-derived lung cancer cell transplantation was performed twenty days before treatment initiation. Raw flow cytometry files were provided by M. Pittet's lab (Massachusetts General Hospital Research Institute, Harvard Medical School, USA). **a-d**) neutrophils were identified as CD45+CD11b+Ly6Cint cells. * $p<0.05$, ** $p<0.01$ from Mann-Whitney test; error bars represent s.e.m. Source data are provided as a Source Data file.

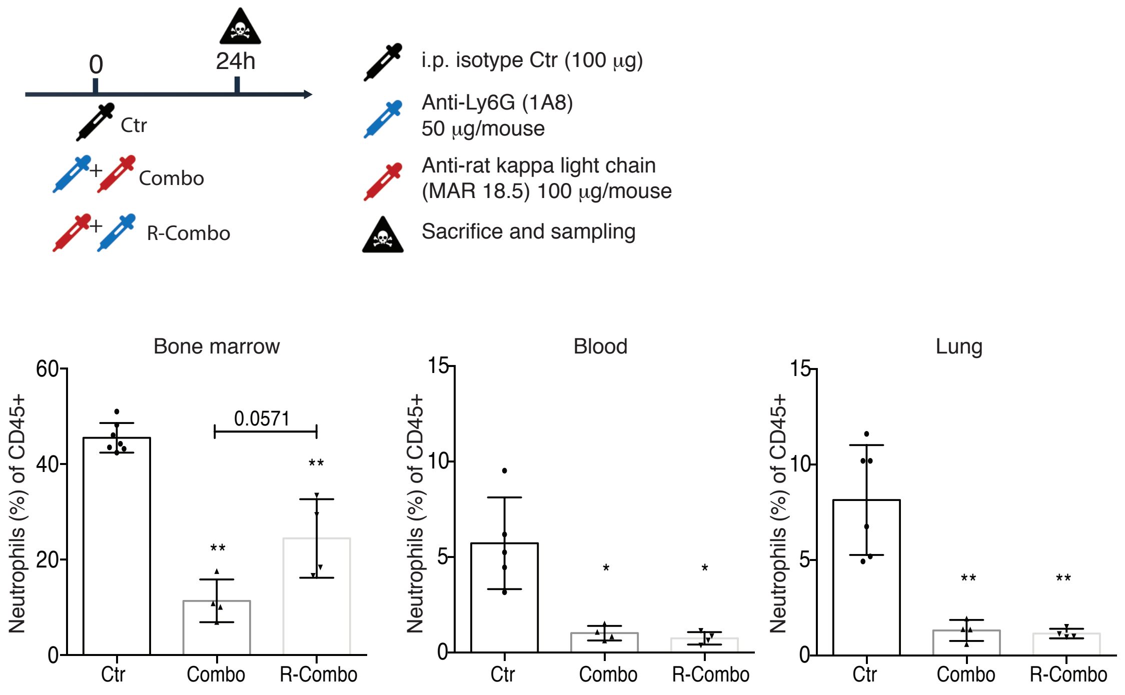

Supplementary Figure 4. Anti-Ly6G manages to find its target despite high amounts of anti-rat antibody. Comparative effect on neutrophil prevalence of anti-Ly6G followed by a secondary anti-rat (Combo) versus a secondary anti-rat followed by anti-Ly6G (Reverse (R)-Combo). Despite the excess of secondary anti-rat, the R-Combo procedure induces neutropenia and confirms the anti-Ly6G manages to find its target at the surface of the cells. Source data are provided as a Source Data file.

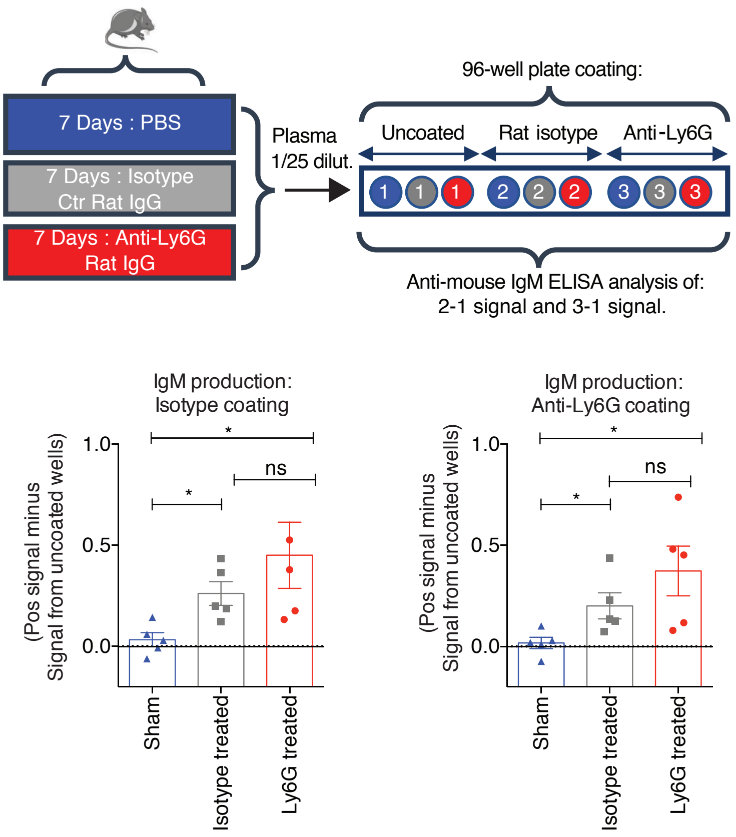

Supplementary Figure 5. Antibody-treated mice develop anti-antibodies specific for the isotype-related heavy chains.
Semi-quantitative ELISAs were performed to detect the occurrence of anti-antibodies in mice treated for 7 days with PBS, isotype control antibody or anti-Ly6G. Each serum sample (at 1/25 dilution) was added into wells loaded with nothing, an isotype control antibody or the anti-Ly6G antibody. Signal intensity of the adequate revelation for antibody-loaded wells was compared to the negative control with no loading. All the treated mice generated anti-antibodies that cross react with both anti-Ly6G and its relevant isotype control, suggesting an immunization specific for the common heavy chains (isotype, non-idiotypic). n=5 mice per group. Each serum sample was equally split and acquired in all three conditions. Source data are provided as a Source Data file.

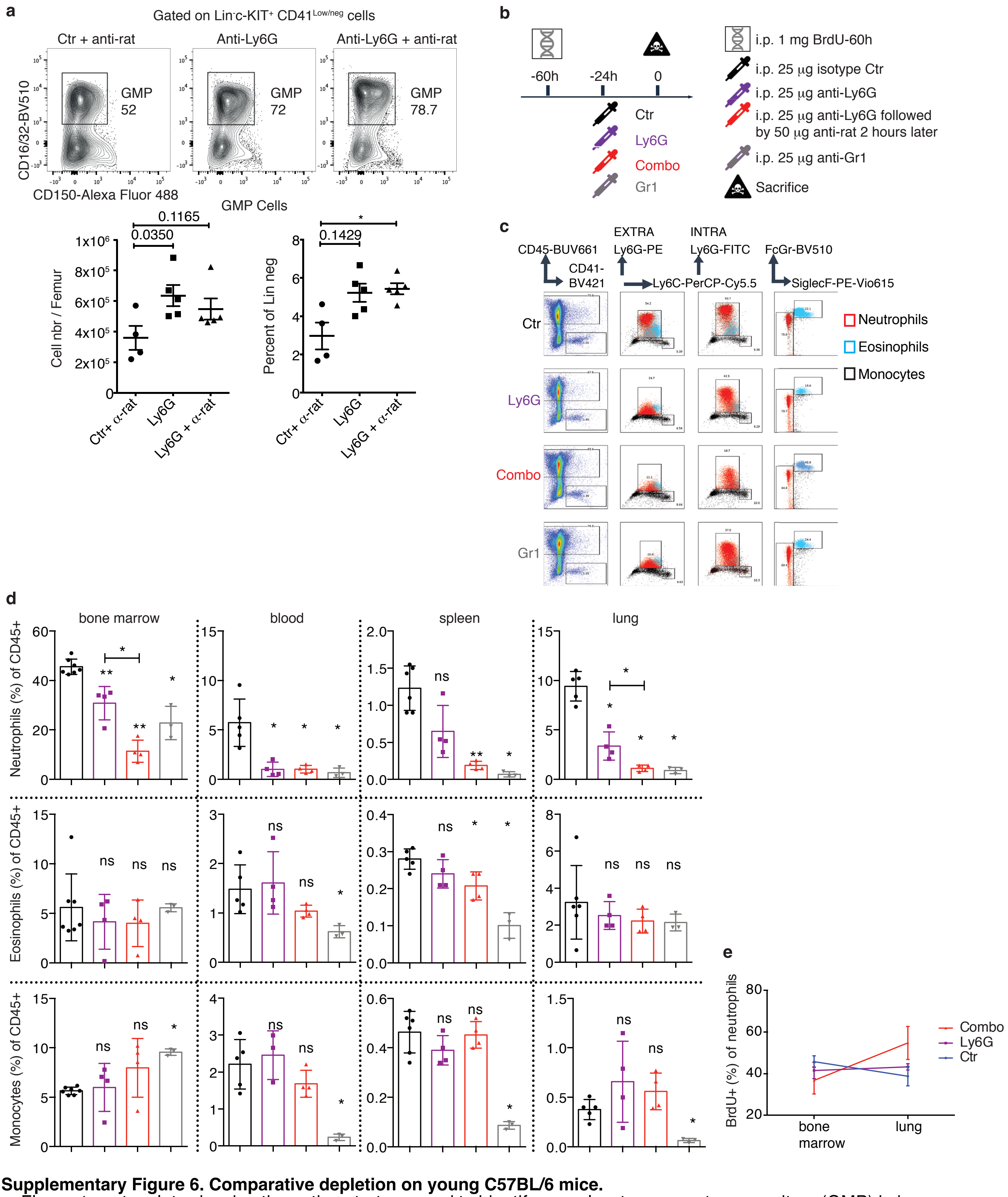

Supplementary Figure 6. Comparative depletion on young C57BL/6 mice.
a. Flow cytometry plots showing the gating strategy used to identify granulocyte-monocyte progenitors (GMP) in bone marrow from young (10-12 weeks) C57BL/6J mice treated with 100 μg anti-Ly6G (day 1), 100 μg anti-rat (day 2), 100 μg anti-Ly6G (day 5) and 50 μg anti-rat (day 6), followed by sacrifice (day 7). Dot plots provide number per femur (left) and percentage (right) of the cells of interest based on Trucount flow cytometry. Lin, Lineage including Ter119, CD19, B220, CD3, CD4, CD8, CD11b, CD11c, NK1.1 and Gr1. n=4-5. **b**. Experimental set-up linked to experiments from Fig. 4 but here performed on young (12 weeks old) C57BL/6 mice and for 1 day of depletion. **c**. Representation of the gating strategy. **d-e**. The prevalence of neutrophils (CD45^{pos}Ly6C^{int}Ly6G^{hi}SiglecF^{neg}FcGr^{int}CD31^{neg}), eosinophils (CD45^{pos}Ly6C^{int}Ly6G^{hi}SiglecF^{pos}FcGr^{hi}CD31^{pos}) and monocytes (CD45^{pos}Ly6C^{hi}Ly6G^{neg}) (**d**) and their flow-rate dynamics using BrdU labelling (**e**) were assessed in the indicated compartments. While anti-Ly6G is as efficient as Combo in this experimental setting, BrdU staining indicates that the cellular flow rate remains stronger in the Combo group. **a**, **d** * $p<0.05$, ** $p<0.01$ from Mann-Whitney test; error bars represent s.e.m. (**a**) or s.d. (**d**). Source data are provided as a Source Data file.

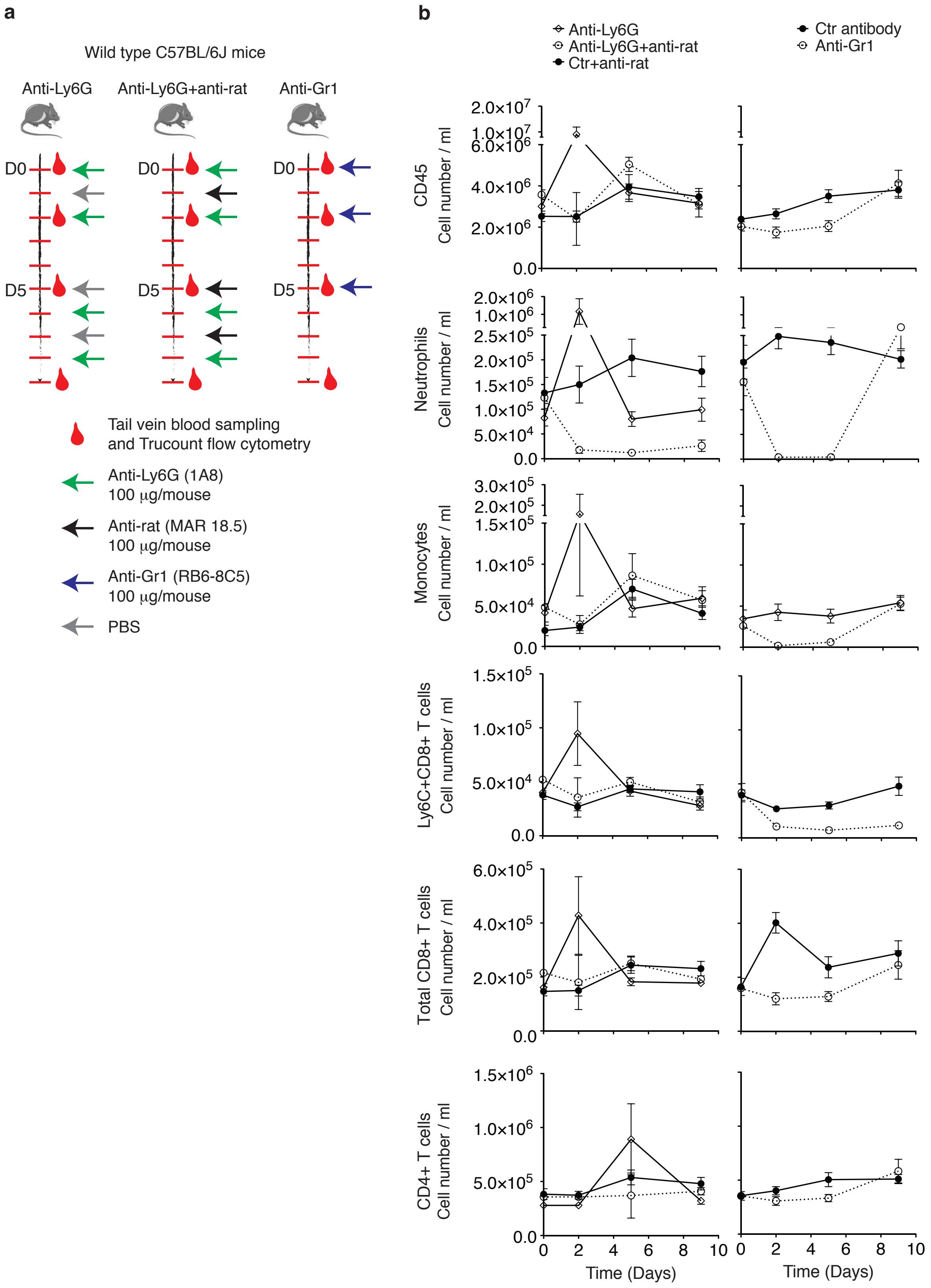

Supplementary Figure 7. Combination of anti-Ly6G and anti-rat IgGκ is specific for neutrophil depletion.
a. Schematic view of the experimental plan. Mice were treated with control or anti-Ly6G, anti-Ly6G plus anti-rat-IgGκ (injection times and amounts represent a non-optimized version of Combo) or anti-Gr1 antibody (100 µg per injection). Anti-Gr1 treatment was stopped at day five. Trucount flow cytometry analyses were performed on 30 µl of tail vein blood samples without red blood cell clearing on every mouse at each indicated time point. **b**. Curves indicate numbers of total immune cells ($CD45^+$), neutrophils ($Ly6C^{int}CD11b^+$), monocytes ($Ly6C^{hi}CD11b^+$), CD8 T cells ($CD3^+CD8^+$), $Ly6C^+$ CD8 T cells ($CD3^+CD8^+Ly6C^+$) and CD4 T cells ($CD3^+CD4^+$). Error bars indicate s.e.m. Source data are provided as a Source Data file.

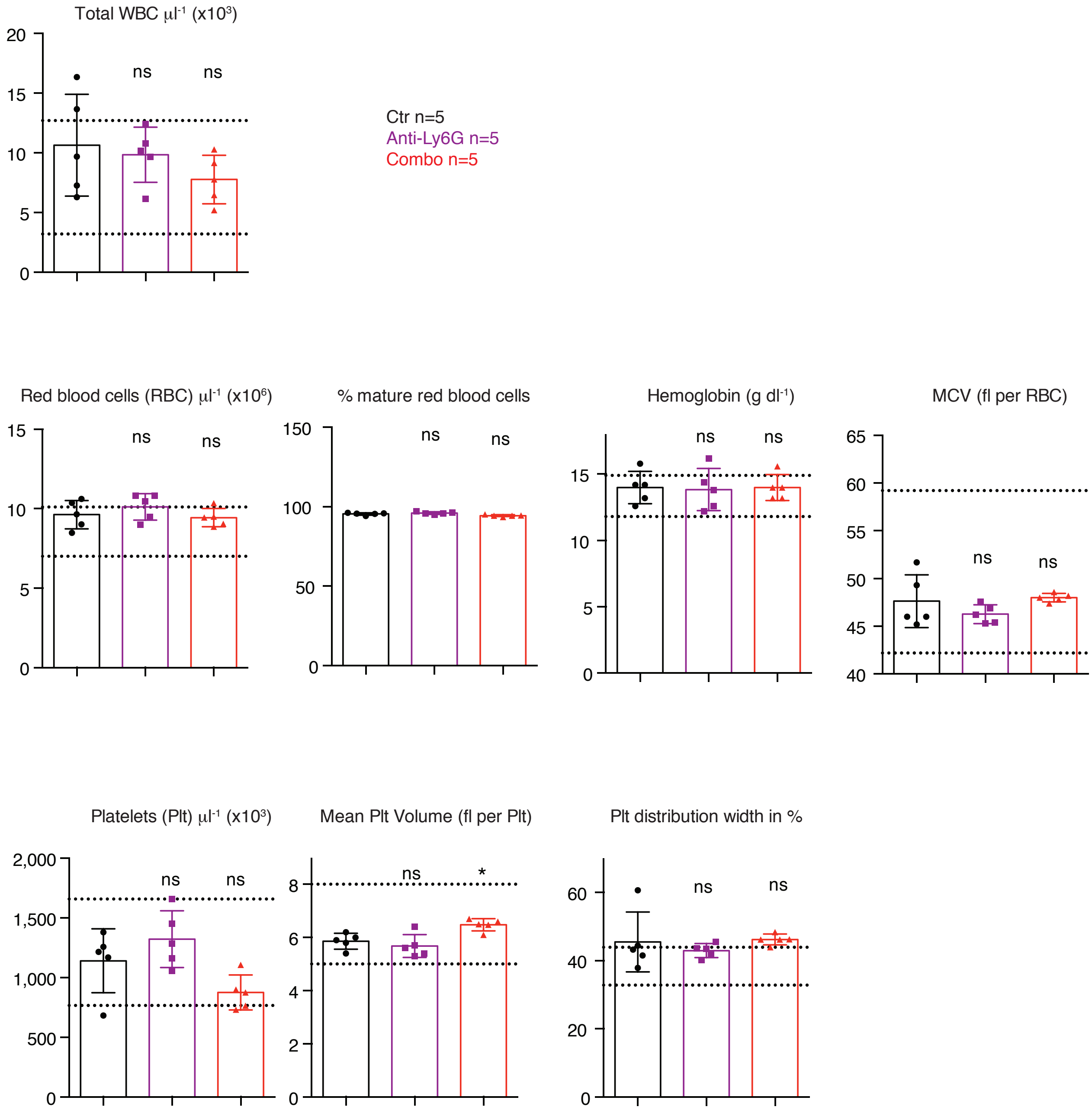

Supplementary Figure 8. There is no broad alteration of blood parameters upon anti-Ly6G or Combo treatment. Blood samples from Fig. 4 were divided before labelling for flow cytometry and a part was acquired on an automated analyzer to exclude any effect on the total white blood cell pool, red blood cell pool or platelet physiology. MCV, mean corpuscular volume; WBC, white blood cell; RBC, red blood cell. Source data are provided as a Source Data file.
