# Supplementary material for: Durable and controlled depletion of neutrophils in mice
Source: Nat Commun. 2020 Jun 2;11:2762. doi: 10.1038/s41467-020-16596-9 (PMC7265525; doi:10.1038/s41467-020-16596-9)
Supplement: Supplementary file 2 — Reporting Summary [file 41467_2020_16596_MOESM2_ESM.pdf]

nature research

Corresponding author(s): Etienne Meylan

Last updated by author(s): Apr 26, 2020

Reporting Summary

Nature Research wishes to improve the reproducibility of the work that we publish. This form provides structure for consistency and transparency in reporting. For further information on Nature Research policies, see Authors & Referees and the Editorial Policy Checklist.

Statistics

For all statistical analyses, confirm that the following items are present in the figure legend, table legend, main text, or Methods section.

n/a	Confirmed	
☐	☒	The exact sample size (*n*) for each experimental group/condition, given as a discrete number and unit of measurement
☐	☒	A statement on whether measurements were taken from distinct samples or whether the same sample was measured repeatedly
☐	☒	The statistical test(s) used AND whether they are one- or two-sided<br>*Only common tests should be described solely by name; describe more complex techniques in the Methods section.*
☒	☐	A description of all covariates tested
☒	☐	A description of any assumptions or corrections, such as tests of normality and adjustment for multiple comparisons
☐	☒	A full description of the statistical parameters including central tendency (e.g. means) or other basic estimates (e.g. regression coefficient) AND variation (e.g. standard deviation) or associated estimates of uncertainty (e.g. confidence intervals)
☒	☐	For null hypothesis testing, the test statistic (e.g. *F*, *t*, *r*) with confidence intervals, effect sizes, degrees of freedom and *P* value noted<br>*Give P values as exact values whenever suitable.*
☒	☐	For Bayesian analysis, information on the choice of priors and Markov chain Monte Carlo settings
☒	☐	For hierarchical and complex designs, identification of the appropriate level for tests and full reporting of outcomes
☒	☐	Estimates of effect sizes (e.g. Cohen's *d*, Pearson's *r*), indicating how they were calculated

Our web collection on statistics for biologists contains articles on many of the points above.

Software and code

Policy information about availability of computer code

Data collection	*Provide a description of all commercial, open source and custom code used to collect the data in this study, specifying the version used OR state that no software was used.*
Data analysis	*Provide a description of all commercial, open source and custom code used to analyse the data in this study, specifying the version used OR state that no software was used.*

For manuscripts utilizing custom algorithms or software that are central to the research but not yet described in published literature, software must be made available to editors/reviewers. We strongly encourage code deposition in a community repository (e.g. GitHub). See the Nature Research guidelines for submitting code & software for further information.

Data

Policy information about availability of data

All manuscripts must include a data availability statement. This statement should provide the following information, where applicable:

- Accession codes, unique identifiers, or web links for publicly available datasets
- A list of figures that have associated raw data
- A description of any restrictions on data availability

No datasets were generated or analysed during the current study.

Field-specific reporting

Please select the one below that is the best fit for your research. If you are not sure, read the appropriate sections before making your selection.

☒ Life sciences ☐ Behavioural & social sciences ☐ Ecological, evolutionary & environmental sciences

Life sciences study design

All studies must disclose on these points even when the disclosure is negative.

Sample size	Based on mouse-to-mouse heterogeneity, groups of 5 to 7 mice per arm were generated.
Data exclusions	There were no data exclusions.
Replication	Several co-authors independently successfully reproduced the methodology and readouts.
Randomization	Mice were randomly spread in all the different groups, when possible different treatment modalities were administrated on mice from the same cage to avoid any potential cage effect.
Blinding	Sample preparation, acquisition and flow cytometry data analyses were performed in a blinded manner.

Reporting for specific materials, systems and methods

We require information from authors about some types of materials, experimental systems and methods used in many studies. Here, indicate whether each material, system or method listed is relevant to your study. If you are not sure if a list item applies to your research, read the appropriate section before selecting a response.

Materials & experimental systems

n/a	Involved in the study	
☐	☒	Antibodies
☒	☐	Eukaryotic cell lines
☒	☐	Palaeontology
☐	☒	Animals and other organisms
☒	☐	Human research participants
☒	☐	Clinical data

Methods

n/a	Involved in the study	
☒	☐	ChIP-seq
☐	☒	Flow cytometry
☒	☐	MRI-based neuroimaging

Antibodies

Antibodies used	APC-Cy7 anti-CD45 eBioSciences 47-0451-82; BUV661 anti-CD45 BD Horizon 565079; PerCP anti-CD45 Miltenyi 130-102-785; BV421 anti-CD41 BioLegend 133912; PE-Vio615 anti-SiglecF Miltenyi 130-112-172; BV711 anti-CD11b BioLegend 101242; PE/Cy7 anti-CD11c BioLegend 117318; BV421 anti-CD11c BioLegend 117330; BV510 anti-CD16/32 BioLegend 101333; BV605 anti-CD62L BioLegend 104437; FITC anti-Ly6G BioLegend 127606; PE anti-Ly6G Biolegend 127608; PE-Cy7 anti-Ly6G Biolegend 127618; DyLight550 Donkey anti-rat Thermofisher SA5-10027; PerCP anti-CD45.2 BioLegend 109826; BV785 anti-CD19 BioLegend 115543; BV510 anti-CD19 BioLegend 115546; BV785 anti-CD4 BioLegend 100552; Alexa Fluor-700 anti-CD4 BioLegend 100536; BV510 anti-CD8a BD Horizon 563068; APC anti-BrdU BioLegend 339808; Alexa Fluor-647 anti-S100A9 BD Pharmingen 565833; APC anti-Gr1 eBioSciences 17-9668-82; FITC anti-Gr1 BioLegend 108406; PE anti-CD31 BioLegend 102408; PE anti-CD3 BioLegend 100308; PerCP-Cy5.5' anti-Ly6C BioLegend 128012; PE anti-CXCR2 BioLegend 149303; Alexa Fluor-488 anti-CD150 BioLegend 115915; BV650 anti-Ly-6A/E BioLegend 108143; BV510 anti-CD16/32 BioLegend 101333; BV421 anti-CD117 BioLegend 105827; PE-Cy7 anti-CD41 BioLegend 133915; Biotin anti-NK1.1 Miltenyi 130-101-888; Biotin anti-CD8a Miltenyi 130-101-956; Biotin anti-CD4 Miltenyi 130-109-412; Biotin anti-TCRb Miltenyi 130-104-809; Biotin anti-Ter119 Miltenyi 130-112-718; Biotin anti-CD19 Miltenyi 130-112-034; Biotin anti-CD45R/B220 Miltenyi 130-110-707.
Validation	All used antibodies are commercially available and catalog number is provided.

Animals and other organisms

Policy information about studies involving animals; ARRIVE guidelines recommended for reporting animal research

Laboratory animals	Most experiments were performed in C57BL/6 mice. However, other strains were used where indicated. Strain name and mouse age are indicated in the Figures.
Wild animals	*Provide details on animals observed in or captured in the field; report species, sex and age where possible. Describe how animals were caught and transported and what happened to captive animals after the study (if killed, explain why and describe method; if released, say where and when) OR state that the study did not involve wild animals.*
Field-collected samples	*For laboratory work with field-collected samples, describe all relevant parameters such as housing, maintenance, temperature, photoperiod and end-of-experiment protocol OR state that the study did not involve samples collected from the field.*
Ethics oversight	All mouse experiments complied with all relevant ethical regulations for animal testing and research. Experiments from the Ecole

Ethics oversight	Polytechnique Fédérale de Lausanne were performed with the ethical approval of the Veterinary Authority of the Canton de Vaud, Switzerland (license number VD2391). Mouse experiments at Massachusetts General Hospital were performed according to approved IACUC guidelines. Mouse experiments performed at the Cancer Research Center of Lyon, Centre Léon Bérard, Lyon, France were approved by the local Animal Ethic Evaluation Committee (CECCAPP: C2EA-15) and authorized by the French Ministry of Education and Research.

Note that full information on the approval of the study protocol must also be provided in the manuscript.

Flow Cytometry

Plots

Confirm that:

☒ The axis labels state the marker and fluorochrome used (e.g. CD4-FITC).

☒ The axis scales are clearly visible. Include numbers along axes only for bottom left plot of group (a 'group' is an analysis of identical markers).

☒ All plots are contour plots with outliers or pseudocolor plots.

☒ A numerical value for number of cells or percentage (with statistics) is provided.

Methodology

Sample preparation	Single cell suspension was obtained after tissue macro-dissection following a protocol described in PMID: 29241546.
Instrument	Acquisitions were performed using the LSRII SORP (Becton Dickinson), a 5-laser and 18-detector analyzer at the EPFL Flow Cytometry Core Facility.
Software	Data analyses were performed using FlowJo X (FlowJo LLC ©).
Cell population abundance	n/a
Gating strategy	Gating strategies are explained in the corresponding Figures or in Supplementary Figure 2.

☒ Tick this box to confirm that a figure exemplifying the gating strategy is provided in the Supplementary Information.
